# O.3.1-1 Relevance of policies to promote physical activity among children and adolescents: the case of Ecuador

**DOI:** 10.1093/eurpub/ckad133.138

**Published:** 2023-09-11

**Authors:** Angélica Ochoa Avilés, Samuel Escandón Dután, Juliana Mejía Grueso, Gisselle Soto, Ximena Vélez, Andrea Ramírez Varela

**Affiliations:** Department of Bioscience, Universidad de Cuenca, Ecuador; Department of Bioscience, Universidad de Cuenca, Ecuador; Research Department, Universidad del Azuay, Ecuador; School of Medicine, Universidad de los Andes, Colombia; angelica.ochoa@ucuenca.edu.ec; Department of Bioscience, Universidad de Cuenca, Ecuador; Research Department, Universidad del Azuay, Ecuador; Research Department, Universidad del Azuay, Ecuador; School of Medicine, Universidad de los Andes, Colombia; angelica.ochoa@ucuenca.edu.ec

## Abstract

**Purpose:**

Physical inactivity among children and adolescents is a public health concern. PA promotion (PAP) policies should rely on the best evidence. This study aimed to contrast PAP policies among children and adolescents in Ecuador with the best available evidence.

**Methods:**

We undertook a comprehensive analysis that included the following procedures: (1) the Country Contact of the Global Physical Activity Observatory GoPA! identified key governmental informants from the Ministries of health, education, and sports at the national and subnational level; (2) the informants provided a list of policies, programs, or technical documents in force that comprise PAP among children and adolescents; (3) the preliminary results of an ongoing umbrella review (PROSPERO ID 408458) were used to identify effective PAP strategies in children and adolescents; (4) the core team determined whether the policies addressed at least one of the eight investments that work for physical activity proposed by the International Society for Physical Activity and Health (ISPAH) (whole-of-school programs, active travel, active urban design, healthcare, public education, including mass media, sport and recreation for all, workplaces, community-wide programs); (5) through a content analysis, we contrasted the strategies proposed in the policy documents with effective strategies identified in systematic reviews.

**Results:**

Seven decision-makers from the health (n = 3), sport (n = 2), and education (n = 2) sectors identified four policies implemented at the national level. One policy addressed the whole-of-school programs approach, while the others the community-wide programs approach. Few strategies in the official documents were aligned with the effective strategies identified in the umbrella review. School-based strategies focused mainly on physical education, and neither the community-based nor the school-based strategies comprehensively addressed the community, school environment, teachers, and parents. In addition, policies in Ecuador lack information regarding an implementation and evaluation plan, the extent, timeframe, and scope of these strategies, and their viability for enhancing PA in children and adolescents.

**Conclusion:**

This study is the basis for adapting and reformulating existing policies in Ecuador to ensure effective and comprehensive PAP in children and adolescents. New studies should analyze the level of implementation and monitor existing and new policies.

**Funding:**

The study was funded by UNICEF-Ecuador.

